# Self-Esteem, Happiness, and Flourishing in Times of COVID-19: A Study During the Lockdown Period in Ecuador

**DOI:** 10.3389/ijph.2022.1604418

**Published:** 2022-02-25

**Authors:** Clara Paz, Carlos Hermosa-Bosano, Paula Hidalgo-Andrade, Javier García-Manglano, Charo Sábada Chalezquer, Claudia López-Madrigal, Cecilia Serrano

**Affiliations:** ^1^ School of Psychology and Education, Universidad de Las Américas, Quito, Ecuador; ^2^ Institute for Culture and Society, University of Navarra, Pamplona, Spain; ^3^ School of Communication, University of Navarra, Pamplona, Spain; ^4^ School of Education and Psychology, University of Navarra, Pamplona, Spain; ^5^ Department of Sociology, Università Cattolica di Milano, Milano, Italy

**Keywords:** COVID-19, Ecuador, flourishing, happiness, self-esteem, well-being

## Abstract

**Objective:** Several studies have investigated the negative toll the pandemic has had on people’s mental health. However, there is limited research on the pandemic’s effect on positive mental health variables. This article reports on the levels of self-esteem and well-being (flourishing and happiness) in a sample of adults living in Ecuador and their relationships with the characteristics of their personal situation and the effects of the COVID-19 pandemic had on their personal lives.

**Methods:** A total of 766 adults completed an anonymous online survey between March and August 2020.

**Results:** Participants reported average scores in the flourishing scale, the majority considered themselves to be happy or very happy people, and more than half presented high levels of self-esteem. Age, education, socioeconomic status, time spent using mobile phones and on hobbies, among others, explained self-esteem, happiness, and flourishing.

**Conclusion:** The relationships between sociodemographic and situational variables of confinement during the pandemic are discussed, as well as the possible predictors of happiness, flourishing, and self-esteem.

## Introduction

The coronavirus pandemic (COVID-19) has altered the lifestyle of many people. Since the beginning of the pandemic, many countries -including Ecuador- adopted various strategies to protect their populations and reduce infections and deaths. These measures included mandatory confinement, curfew, mobility restrictions, teleworking, remote education, mandatory use of masks, limitation of capacity in restaurants and commercial spaces, restrictions in bars and nightlife centers, among others [1].

Evidence indicates that the longer people are in isolation or confinement, the greater the negative effects on their mental health [2]. Several studies in Ecuador [3, 4] and around the world [5, 6] report these detrimental effects on the mental health of people because of the pandemic. Most psychological research has focused on exploring the negative consequences of restrictive measures, including symptoms of stress, psychological distress, confusion, and anger [2].

However, to gain a better understanding of people’s well-being and mental health during this health crisis, it is imperative to analyze its potential effects on other aspects of the health spectrum, such as the levels of self-esteem, happiness, and flourishing. Self-esteem is positively related to general well-being and negatively related to stress, anxiety, and depression [7]. Studies during the COVID-19 pandemic have found that self-esteem could be an anxiety-buffer which can also protect from the impact of other factors, such as depression and loneliness [8].

Another relevant construct in positive psychology is happiness. Happiness allows noticing and taking advantage of positive behaviors and circumstances [9] and relates to better mental and physical health [10]. Studies conducted during the pandemic show correlations between happiness and other well-being variables. For example, a longitudinal study found that a decrease in happiness predicted a decrease in perceived health and was associated with a decreased perceived quality of life [11].

Moreover, it is known that the mere absence of illness is neither a determinant of well-being nor an indicator of good mental health [12]. The presence of mental health is necessary to attain integral well-being, which has been defined as flourishing or prosperity. Despite some differences in its definition, a flourished person is characterized for having high levels of subjective well-being [13] and for being functional, both psychologically and socially, through positive relationships, meaning in life, self-acceptance, and self-esteem [14]. During the pandemic, a study showed that there was a negative relationship between COVID-19 fear and human flourishing because of self-isolation and restricted activities, particularly during the lockdown periods [15].

This article seeks to report the levels of self-esteem and well-being (flourishing and global happiness) experienced by people living in Ecuador during the months of quarantine due to the COVID-19 pandemic. We consider that self-esteem, global happiness, and flourishing may act as protective factors against the possible negative consequences of the pandemic. Also, understanding how sociodemographic characteristics and personal situations are related to these variables will allow the design and implementation of effective interventions or other kinds of support from a positive psychology perspective.

## Methods

### Design and Participants

This article analyzes data from 766 individuals who reported being in Ecuador at the time of the study (March to August 2020). This research is part of a non-experimental, cross-sectional, quantitative study carried out in 11 Spanish-speaking countries (Welfare in quarantine: https://en.unav.edu/web/culture-and-society-institute/youth-in-transition/research/bienestar-cuarentena-coronavirus-tecnologia). To participate, individuals had to be 18 years old or older, indicate the country in which they were living during the lockdown, and complete an anonymous online survey.

### Instruments

The following information was analyzed in this study:

#### Sociodemographic Data

This section included *s*elf-report questions on sex, age, highest level of education, as well as employment, socioeconomic, marital, and relationship status (i.e., with or without a steady partner).

#### Personal Living Conditions During COVID-19

This section inquired about the number of people with whom the respondent was living in the same household and how many of them were older than 60 years or younger than 12 years of age. We also inquired about whether the respondent was in the highest risk group for COVID-19 (due to age or pre- existing illness), whether there were changes in his/her work situation due to the pandemic (loss of a job or not), the number of weekly outings during the quarantine, and the perceived increase in time spent using cell phones, doing hobbies, and conducting physical exercise.

#### COVID-19 Effects

This section included questions on whether the respondent, or a person close to them had had symptoms, had received a positive diagnosis, had been hospitalized, or had died from COVID-19.

#### Self-Esteem Scale

We used the Rosenberg Scale [16], validated in Spanish [7] in this study. This questionnaire consists of 10 items using a Likert-type scale from 1 = not at all to 4 = very much. Some examples of its statements are “I feel that I’m a person of worthy, at least on an equal plane with others” and “On the whole, I am satisfied with myself.” After reversing five items and summing the total score, responses are classified into low [10–25], medium [26–29], and high (30–40) levels of self-esteem. In this study, this scale showed a reliability of 0.85.

#### Global Happiness

To explore perceived global happiness, respondents were asked: “Do you consider yourself a happy person?” Response options ranged from 1 = not at all to 4 = very much.

#### Flourishing

The Flourishing Scale [17] was used in its Spanish version [18]. This scale assesses social and psychological flourishing through the perception of success in relationships, self-esteem, purpose in life, and optimism. It has eight items with Likert-type response options (1 = not at all to 4 = very much). The overall score indicates the psychological well-being corresponding to flourishing, and it is obtained from the sum of all responses. The total score ranges from 8 to 32. The higher the total score, the higher the levels of well-being. Cronbach’s alpha of this scale was 0.89.

### Procedure

The study received approval from the ethics committee of the University of Navarra (2020.087) before data collection. The survey was created in *Google Forms* and the link was distributed through the social networks of the researchers and the research teams involved; an email was also sent through the distribution lists of the university of the research group in Ecuador, after obtaining the respective permissions. The informed consent protocol, specifying the details of the research and the voluntariness and anonymity of participation, was shown on the first page of the survey. Data were collected during the highest peak of the first wave of the COVID-19 pandemic in Ecuador between March and August 2020.

### Data Analysis

Descriptive analyses were performed for the sample and the study variables. Pearson’s correlations were run for age, number of people in the same household, number of people over 60 and under 12 years old living in the same household. In addition, according to the type of variable, different analyses were performed to determine the univariate relationships and differences on the study variables (self-esteem, happiness, and flourishing) based on the sociodemographic variables (sex, higher level of education achieved, employment, socioeconomic, marital, and relationship status), the situational context of confinement (whether the respondent was in the highest risk group for COVID-19, whether the participant had lost his/her job, the number of weekly outings, and the perceived increase in time spent using cell phones, hobbies, and physical exercise), and the effects of COVID-19 (whether the respondent or a close person had symptoms, had a positive diagnosis, had been hospitalized, or had died from COVID-19). Given that self-esteem and happiness were codified by levels, Chi-squares were performed to identify potential differences. Due to its continuous nature, ANOVAs were conducted when analyzing the flourishing scores. Finally, for each of the three main variables, linear regressions were performed placing them as dependent variables and including, as independent variables, those that were shown to be significant in the previous analyses. All analyses were carried out using SPSS version 25.

## Results

### Sample Characteristics

Of the total sample (*N* = 766), 274 were men and 492 women. Participants’ age ranged from 18 to 85 years (*M* = 32.35, *SD* = 28.0). Table 1 shows their sociodemographic details.

**TABLE 1:** Sociodemographic data of the sample (N = 766) Self-esteem, happiness and flourishing in times of COVID-19: A study during the lockdown period in Ecuador, Ecuador, 2020.

	*n*	%
Sex		
Men	274	35.8
Women	492	64.2
Age (by groups)		
18–22	193	25.2
23–29	219	28.6
30–39	153	20.0
40–49	98	12.8
50–59	76	9.9
Over 60	27	3.5
Education		
High school diploma or less	203	26.5
Professional or technical training	83	10.8
Higher education	480	62.7
Employment status		
Study	233	30.4
Study and work	126	16.4
Work	257	33.6
Unemployed	88	11.5
Retired	13	1.7
Other	49	6.4
Socioeconomic status		
Low	92	12.0
Medium	435	56.8
High	239	31.2
Marital status		
Single with no children	447	58.4
Single with children	36	4.7
Separated or divorced	50	6.5
Widower	4	0.5
Married or in a civil union	229	29.9
Couple situation		
Without a stable partner	537	70.1
With a stable partner	229	29.9

Most participants (*n* = 649, 84.7%) reported spending the lockdown period with others in the same household. The number of people they were living with ranged from 1 to 14 (*Mdn* = 3, *IQR* = 2). Of the total number of respondents who indicated living with people over 60 years (*n* = 297, 38.8%), the majority reported living with one or two people. On the other hand, of the participants who lived with children under 12 years (*n* = 235, 30.7%), 160 (20.9%) lived with one child, 52 (6.8%) with two children, and 23 (3.0%) with three children. Table 2 shows the details of participants’ personal living conditions.

**TABLE 2:** Personal situations during the quarantine by COVID-19. Self-esteem, happiness and flourishing in times of COVID-19: A study during the lockdown period in Ecuador, Ecuador, 2020.

	*n*	%
Impact of COVID-19 on job stability		
Lost job	41	5.4
Did not lose the job	725	94.6
Weekly times leaving the house during quarantine		
None	308	40.2
Once or twice	374	48.8
Three or more times	84	11.0
Increased use of cellular phones		
Nothing	18	2.3
Little	186	24.3
Very	422	55.1
Much	140	18.3
Changes in the time dedicated to physical exercise		
It has decreased	349	45.6
It has remained the same	209	27.3
It has increased	208	27.2
Changes in time spent on hobbies		
It has decreased	281	36.7
It has remained the same	205	26.8
It has increased	280	36.6
Person at risk due to age or previous pathology		
Yes	119	15.5
No	647	84.5
Cohabitation with other people		
Yes	649	84.7
No	117	15.3
Cohabitation with people over 60 years of age		
Yes	297	38.8
No	469	61.2
Cohabitation with children under 12 years of age		
Yes	235	30.7
No	531	69.3

In addition, as seen in Table 3, most participants reported having family members or acquaintances who had experienced symptoms of the disease, or who had received a positive diagnosis. However, most participants reported not knowing whether people in their social network had been hospitalized or had died from COVID-19.

**TABLE 3:** People affected by COVID-19. Self-esteem, happiness and flourishing in times of COVID-19: A study during the lockdown period in Ecuador, Ecuador, 2020.

	With symptoms	With diagnosis	Hospitalized	Deceased
	*n* (%)	*n* (%)	*n* (%)	*n* (%)
Respondent	99 (12.9)	64 (8.4)	4 (0.5)	—
Family	535 (69.8)	511 (66.7)	49 (6.4)	32 (4.2)
Acquaintances	98 (12.8)	144 (18.8)	181 (23.6)	146 (19.1)
Nobody	34 (4.4)	47 (6.1)	532 (69.5)	588 (76.8)

### Key Variables: Self-Esteem, Happiness, and Flourishing

We analyzed the potential relationships between the variables of interest and the sociodemographic, situational, and pandemic-related variables. Table 4 indicates the levels of self-esteem, global happiness, and flourishing according to the sociodemographic variables and Table 5 shows these key variables according to the context variables.

**TABLE 4:** Self-esteem, happiness, and flourishing according to sociodemographic variables. Self-esteem, happiness and flourishing in times of COVID-19: A study during the lockdown period in Ecuador, Ecuador, 2020.

	*n*	Self-esteem			Consideration as a happy person				Flourishing
		Low	Medium	High	Nothing	Little	Very	Much	*M* (*SD*)
		%	%	%	%	%	%	%	
Total	766	18.4	22.3	59.3	1.3	16.8	59.1	22.7	6.89 (1.82)
Sociodemographics									
Sex									
Male	274	6.79	22.26	60.95	2.19	15.33	58.03	24.45	6.86 (1.81)
Female	492	19.31	22.36	58.33	0.81	17.68	59.76	21.75	6.91 (1.83)
Age (by groups)									
18–22	193	29.53	26.94	43.52	2.07	20.73	61.14	16.06	6.63 (1.82)
23–29	219	20.55	18.26	61.19	0.91	18.72	57.53	22.83	7.03 (1.88)
30–39	153	14.38	29.41	56.21	1.31	18.95	59.48	20.26	6.74 (1.82)
40–49	98	10.20	12.24	77.55	2.04	11.22	53.06	33.67	7.09 (1.75)
50–59	76	7.89	22.37	69.74	0.00	6.58	65.79	27.63	703 (1.65)
Over 60	27	3.70	18.52	77.78	0.00	11.11	59.26	29.63	7.44 (1.83)
Education									
High school diploma or less	203	29.06	27.59	43.35	2.96	19.70	59.11	18.23	6.59 (1.84)
Professional or technical training	83	13.25	19.28	67.47	0.00	13.25	65.06	21.69	6.73 (1.65)
Higher education	480	14.79	20.63	64.58	0.83	16.25	58.13	24.79	7.05 (1.82)
Employment status									
Study	233	27.90	24.46	47.64	1.72	21.46	62.66	14.16	6.71 (1.8)
Study and work	126	15.08	15.87	69.05	1.59	13.49	60.32	24.60	7.19 (1.79)
Work	257	11.28	21.01	67.70	0.39	13.62	57.20	28.79	7.14 (1.72)
Unemployed	88	19.32	27.27	53.41	3.41	19.32	59.09	18.18	6.56 (1.99)
Retired	13	0.00	23.08	76.92	0.00	0.00	38.46	61.54	7.98 (1.39)
Other	49	22.45	26.53	51.02	0.00	20.41	55.10	24.49	6.0 (1.74)
Socioeconomic status									
Low	92	33.70	26.09	40.22	1.09	31.52	57.61	9.78	6.08 (1.78)
Medium	435	16.55	21.84	61.61	1.38	16.09	58.85	23.68	6.9 (1.8)
High	239	15.90	21.76	62.34	1.26	12.55	60.25	25.94	7.18 (1.78)
Marital status									
Single with no children	447	24.16	22.37	53.47	1.79	19.02	59.06	20.13	6.82 (1.85)
Single with children	36	13.89	30.56	55.56	2.78	22.22	55.56	19.44	6.6 (1.93)
Separated or divorced	50	10.00	20.00	70.00	0.00	20.00	52.00	28.00	6.6 (1.61)
Widower	4	0.00	0.00	100.00	0.00	0.00	75.00	25.00	7.5 (1.48)
Married or with a stable partner	229	10.04	21.83	68.12	0.44	11.35	61.14	27.07	7.13 (1.77)
Couple situation									
Without a stable partner	537	21.97	22.53	55.49	1.68	19.18	58.29	20.86	6.79 (1.83)
Married or with a stable partner	229	10.04	21.83	68.12	0.44	11.35	61.14	27.07	7.13 (1.77)

**TABLE 5:** Self-esteem, happiness, and flourishing according to personal situation during quarantine and effects of COVID-19. Self-esteem, happiness and flourishing in times of COVID-19: A study during the lockdown period in Ecuador, Ecuador, 2020.

	*n*	Self-esteem			Consideration as a happy person				Flourishing
		Low	Medium	High	Nothing	Little	Very	Much	*M* (*SD*)
		%	%	%	%	%	%	%	
Total	766	18.4	22.3	59.3	1.3	16.8	59.1	22.7	6.89 (1.82)
Personal situation during quarantine by COVID-19									
Impact of COVID-19									
Lost job	725	18.34	22.07	59.59	1.10	16.55	59.59	22.76	6.92 (1.8)
Did not lose the job	41	19.51	26.83	53.66	4.88	21.95	51.22	21.95	6.37 (2.03)
Weekly times leaving the house during quarantine									
None	308	21.10	20.78	58.12	0.97	18.51	57.47	23.05	7.06 (1.9)
Once or twice	374	18.45	22.46	59.09	1.34	16.31	59.09	23.26	6.80 (1.81)
Three or more times	84	8.33	27.38	64.29	2.38	13.10	65.48	19.05	6.69 (1.5)
Increased use of cellular phones									
Nothing	18	22.22	27.78	50.00	0.00	27.78	55.56	16.67	7.11 (1.83)
Little	186	13.98	20.97	65.05	0.54	15.59	60.22	23.66	6.97 (1.8)
Very	422	17.54	21.56	60.90	1.42	15.17	62.56	20.85	6.84 (1.47)
Much	140	26.43	25.71	47.86	2.14	22.14	47.86	27.86	6.93 (2.07)
Changes in time spent exercising									
It has decreased	349	19.20	22.92	57.88	2.01	20.63	55.87	21.49	6.74 (1.92)
It has remained the same	209	18.18	20.57	61.24	0.96	14.35	60.29	24.40	7.08 (1.71)
It has increased	208	17.31	23.08	59.62	0.48	12.98	63.46	23.08	6.96 (1.74)
Changes in time spent on hobbies									
It has decreased	281	23.13	22.42	54.45	1.07	23.49	56.94	18.51	6.65 (1.86)
It has remained the same	205	16.59	25.85	57.56	1.95	12.68	63.41	21.95	6.85 (1.79)
It has increased	280	15.00	19.64	65.36	1.07	13.21	58.21	27.50	7.17 (1.77)
Person at risk due to age or previous pathology									
Yes	119	11.76	20.17	68.07	0.84	17.65	53.78	27.73	6.95 (1.89)
No	647	19.63	22.72	57.65	1.39	16.69	60.12	21.79	6.88 (1.81)
Cohabitation with other people									
Yes	649	19.26	22.50	58.24	1.39	17.57	57.32	23.73	6.88 (1.83)
No	117	13.68	21.37	64.96	0.85	12.82	69.23	17.09	6.93 (1.79)
Cohabitation with people over 60 years of age									
Yes	297	20.20	24.24	55.56	1.68	19.87	56.57	21.89	6.79 (1.83)
No	469	17.27	21.11	61.62	1.07	14.93	60.77	23.24	6.96 (1.81)
Cohabitation with children under 12 years of age									
Yes	235	19.15	22.98	57.87	2.55	17.87	54.47	25.11	6.82 (1.99)
No	531	18.08	22.03	59.89	0.75	16.38	61.21	21.66	6.93 (1.74)
People, close to respondent, affected by COVID-19									
With symptoms									
Respondent	99	20.20	24.24	55.56	1.01	18.18	58.59	22.22	6.98 (1.87)
Family	535	18.69	19.81	61.50	1.68	15.33	60.37	22.62	7.05 (1.83)
Known	98	13.27	28.57	58.16	0.00	19.39	55.10	25.51	6.49 (1.61)
Nobody	34	23.53	38.24	38.24	0.00	29.41	52.94	17.65	5.64 (1.39)
With diagnosis									
Respondent	64	20.31	28.13	51.56	0.00	17.19	62.50	20.31	6.83 (1.75)
Family	511	18.98	20.16	60.86	1.96	15.66	60.86	21.53	7.05 (1.81)
Known	144	13.19	24.31	62.50	0.00	17.36	54.17	28.47	6.74 (1.82)
Nobody	47	25.53	31.91	42.55	0.00	27.66	51.06	21.28	5.68 (1.50)
Hospitalized									
Respondent	4	25.00	75.00	0.00	0.00	50.00	50.00	0.00	5.0 (1.36)
Family	49	16.33	18.37	65.31	0.00	18.37	55.10	26.53	7.22 (1.89)
Known	181	14.36	22.10	63.54	0.55	13.26	56.91	29.28	7.01 (1.89)
Nobody	532	19.92	22.37	57.71	1.69	17.67	60.34	20.30	6.84 (1.78)
Deceased									
Family	32	18.75	21.88	59.38	0.00	21.88	56.25	21.88	7.21 (1.96)
Known	146	13.70	22.60	63.70	0.68	12.33	56.16	30.82	6.99 (1.92)
Nobody	588	19.56	22.28	58.16	1.53	17.69	60.03	20.75	6.85 (1.79)

### Self-Esteem

On average, the sample presented high levels of self-esteem (*M* = 30.3, *SD* = 5.4). When analyzing self-esteem by categorial levels, results shown that slightly more than half of the sample (*n* = 454, 59.3%) presented high levels of self-esteem, whereas the rest presented medium (*n* = 171, 22.3%) and low (*n* = 141, 18.4%) levels. When analyzing its relationship to the sociodemographic variables, sex was not significant, but age was positively correlated (*r* = 0.22, *p* < 0.001). It was observed that people between 18 and 22 years of age had lower self-esteem than the rest of the age groups, χ^*2*^ (10, *N* = 766) = 54.67, *p* < 0*.*001. A significant relationship was also observed with the education level. People with high school education or less (*n* = 203) had lower levels of self-esteem (29.06% low, 27.59% medium and 43.35% high) than those with professional or technical training (*n* = 83; 13.25% low, 19.28% medium and 67.47% high) and those with higher education (*n* = 480; 14.79% low, 20.63% medium and 64.58% high), χ^*2*^ = (4, *N* = 766) = 32.52, *p* < 0.001.

In the case of employment status, people who reported only studying had significantly lower levels of self-esteem compared to those who worked and studied, and those who reported only working, χ ^*2*^ (10, *N* = 766) = 38.68, *p* < 0.001. In contrast, people with a low socioeconomic status presented significantly lower levels of self-esteem compared to people with high and medium levels, χ ^*2*^ (4, *N* = 766) = 20.39, *p* < 0.001.

Regarding marital and relationship status, it was observed that single people without children, χ ^*2*^ (8, *N* = 766) = 29.67, *p* < 0.001, and those without a stable partner, χ ^2^ (2, *N* = 766) = 16.77, *p* < 0*.*001, had significantly lower levels of self-esteem compared to married people or those with a stable partner.

When analyzing participants’ context during COVID-19, only the perceived changes in cell phone use and changes in the amount of time spent on hobbies showed significant relationships with self-esteem. People who claimed to have increased their cell phone usage time by “a lot” presented lower levels of self-esteem compared to those who increased their usage by “a little” and “quite a lot” χ ^*2*^ (6, *N* = 766) = 12.99, *p =* 0.04. People who reported having decreased their time dedicated to hobbies also showed significantly lower levels of self-esteem compared to those who reported an increase on it, χ ^*2*^ (4, *N* = 766) = 10.54, *p* = 0.03. On the other hand, knowing people directly affected by COVID-19 or presenting these effects oneself (having symptoms, confirmed diagnosis, hospitalization) did not have statistically significant relationships.

Finally, we conducted a linear regression using variables that were significantly associated with self-esteem as independent factors. After performing the first regression model, the variables marital status (*β* = −0.014, *p* = 0.89), sentimental situation (*β* = 0.043, *p* = 0.97) and cell phone use (*β* = −0.057, *p* = 0.11) were excluded. The model that significantly explained self-esteem [*F* (5, 760) = 14.54, *p* < 0.001, *R* ^*2*^_*adjusted*__*=*0*.*_081] included age (*β* = 0.164, *p <* 0.001), education (*β* = 0*.*103, *p* = 0.007), employment status (*β* = −0.084, *p* = 0.02), socioeconomic status (*β* = 0.098, *p* = 0.005), and time spent in hobbies (*β* = 0.095, *p* = 0.007).

### Global Happiness

Most respondents considered themselves to be happy people. Responses on this variable ranged from not at all (*n* = 10, 1.3%), a little (*n* = 129, 16.8%), quite a lot (*n* = 453, 59.1%), and very much (*n* = 174, 22.7%). The sociodemographic variables that showed significant relationships with global happiness levels were age (*r* = 0.15, *p* < 0.001), employment status (χ ^2^ (15, *N* = 766) = 37.21, *p* = 0*.*001), socioeconomic status (χ ^2^ (*6,* N = 766) = 22.72, *p* = 0*.*001) and sentimental status (χ ^2^ (*3*, *N* = 766) = 10.68, *p* = 0*.*01). People who worked and those who were retired reported being happier than those who were studying. Likewise, retired people reported being significantly happier than unemployed people. On the other hand, people with high and medium socioeconomic status reported significantly higher levels of happiness than people with lower socioeconomic status. Finally, married people and those with a stable partner considered themselves happier than people without a stable partner.

Regarding the personal living conditions’ variables, only the time dedicated to hobbies was significantly related, χ ^2^ (6, *N* = 766) = 18.53, *p* = 0.005. People who had decreased their time engaged in hobbies during lockdown presented lower overall happiness compared to those who had increased it.

When performing a linear regression to predict happiness levels, all variables significantly related were included as predictors. Employment (*β* = −0.050, *p* = 0.16) and sentimental status (*β* = 0.043, *p* = 0.30) were excluded. The model that significantly explained happiness [*F* (3, 762) = 5.76, *p* < 0.001*, R* ^*2*^_*adjusted*__*=*_0*.*047] included age (*β = 0*.140, *p <* 0.001), socioeconomic status (*β* = 0*.*122, *p* = 0.001), and time spent in hobbies (*β* = 0*.*111, *p* = 0.*002*).

### Flourishing

The sample obtained average levels in the flourishing scale (*M* = 6.89, *SD* = 1.82). Flourishing and age had a positive correlation (*r* = 0.07, *p =* 0*.*04). Also, people with higher education showed significantly higher levels of flourishing than those with high school education or less [*F* (2,763) = 5.07, *p =* 0.006]. Similarly, people who described their work situation as “other” reported significantly lower levels of flourishing compared to those who are both studying and working and only working [*F* (5,760) = 6.07, *p* < 0.001]. People with a low socioeconomic status presented lower levels of flourishing than those in high and medium levels [*F* (2,763) = 12.58, *p* < 0.001]. Finally, people without a stable partner indicated lower levels of flourishing than those who were married or had a stable partner [*F* (1,764) = 5.81, *p* = 0.02].

Concerning the personal living conditions during lockdown, although the student’s *t-test* was not significant between living alone or not, the number of people living in the same household showed a significant negative correlation with flourishing (*r* = −0.08, *p* = 0.02). There were lower levels of flourishing when participants reported living with more people. On the other hand, people who had decreased the time spent on their hobbies presented lower levels of flourishing than those who had increased it [*F* (2,763) = 5.91, *p* = 0.003].

When analyzing the levels of flourishing according to the COVID-19 effects’ variables, the results indicated that people who did not know anyone with symptoms presented lower levels of flourishing than those who had a family member with symptoms or those who themselves presented symptoms. Similarly, people who had a family member with symptoms had higher levels of flourishing than those who knew someone (a non-family member) in that situation [*F* (3,762) = 8.47, *p* < 0.001]. Regarding COVID-19 diagnosis, people who did not know anyone with a confirmed diagnosis had significantly lower levels of flourishing compared to those who had a family member, an acquaintance, or those who had the diagnosis themselves [*F*(3,762) = 8.89, *p* < 0.001]. Knowing someone who had required hospitalization or who had died was not significant related to the levels of flourishing.

Finally, when running the linear regression, non-significant variables were excluded, age (*β* = 0.021, *p* = 0.62), education (*β* = 0.059, *p* = 0.13), sentimental situation (*β* = 0.052, *p* = 0.20), number of people living in the same household (*β* = −0.051, *p* = 0.15), and COVID-19 symptoms (*β* = −0.034, *p* = 0.56). The model that significantly explained flourishing [*F* (4, 761) = 16.33, *p* < 0.001, *R* ^*2*^_*adjusted*__*=*_0*.*074] included employment status (*β =* −0.140, *p <* 0.001), socioeconomic status (*β* = 0*.*147, *p* < 0.001), time spent in hobbies (*β* = 0.*113*, *p* = 0.001), and knowing a person with a diagnosis of COVID-19 (*β* = −146, *p* < 0.001)*.*

## Discussion

This study analyzed the self-esteem and the well-being (flourishing and happiness) of people living in Ecuador during lockdown due to the COVID-19 pandemic. Like studies conducted before the pandemic in the Ecuadorian population, our sample presented high levels of self-esteem. For example, one conducted in 2017 [19] found similar overall levels of self-esteem (*M* = 31.7) to those found in our study (*M* = 30.3), and highlighted self-esteem as a protective factor against meeting the criteria for a major depressive episode, panic disorder, and suicide risk [19].

Regarding happiness, unlike studies that report a decrease in happiness and optimism [20] and similar to others in the same cultural context [21], most respondents in our study considered themselves to be happy people. This result is not surprising given that the 2021 World Happiness Report shows an increase in sadness and worry globally, but an stable overall life evaluations and happiness ranking [22]. Also, according to the World Happiness Index, in 2020 Ecuador ranked in 58th place of the 149 countries [23].

Unlike a cross-sectional study in the United States that found that COVID-19 fear significantly decreased human flourishing and that gender moderated the relationship between those two variables [15], people from our sample showed average levels of flourishing. Longitudinal research may also support our findings. For example, a study that evaluated differences in mental health before and during the pandemic showed that, although there was a decline in mental health indicators -including flourishing- during the initial stages of the lockdown measures, they were not significant when compared to the changes from previous years [24]. Maybe, during the pandemic, people had to adapt and learn to manage their circumstances and achieved environmental mastery and personal growth, characteristics related to flourishing [25]. Research also suggests that people who experience this growth, highlight the opportunities that may arise during life transitions [25]. Results from the current study could show similar pathways.

Regarding sociodemographic and context variables, research shows that there is no definitive evidence linking them as risk factors for mental health problems during quarantine [2], although some have been found that being younger, having lower levels of education, being female, and having a child [26] are features related to greater psychological distress.

The results of our study can be contrasted with others conducted in previous quarantines and with current others about psychological distress that indicate that younger people have higher levels of stress, anxiety, and depression [27]. In concordance with this body of research, we found that the older the age, the higher the self-esteem, the greater the overall happiness, and the higher the levels of flourishing. On the other hand, lower levels of education were related to lower self-esteem and lower levels of flourishing. Likewise, people with low socioeconomic status had significantly lower levels of self-esteem, happiness, and flourishing. Such results highlight the importance of the socio-cultural determinants of health and the importance of shifting our perspective to see the COVID-19 situation as a syndemic rather than a pandemic [28]. This way, we could consider the interaction between social and biological factors to better face this disease and its consequences.

Single people without children and those without a stable partner presented lower self-esteem. The latter also considered themselves to be less happy and present lower levels of flourishing. Concerning occupational status, people who were only studying presented lower self-esteem and overall happiness than those who had a job. This finding complements another study [29] where students presented greater psychological distress. A possible explanation for this result may be that the change of study modality (from face-to-face to virtual) confronted students with a lifestyle in which not only their social contact was modified but also their access to education, with institutions and teachers not fully prepared for this abrupt change.

Although social contact is important, the results indicated that there are lower levels of flourishing when more people live in the same physical space. This may be due to the long duration of confinement and restrictions in the country. In addition, it can be attributed to the fact that being in the same household with many people means sharing spaces and resources without the possibility of external distractions or time away from home, which can result in the emergence of interpersonal conflicts. Thus, being in the same physical space might not translate into having social support or positive social interactions.

During this pandemic, there has been an insistence on voluntary confinement, mandatory lockdowns, and physical distancing to curb contagion. This shift towards carrying out daily activities remotely encouraged greater use of technology. However, we found that people who increased the time they spent using cell phones had lower levels of self-esteem than those who perceived that they were using them the same amount of time as before the pandemic. Although the Internet can be a convenient way to stay in touch with others, excessive use may have more negative than positive effects. This result is in line with several previous studies that relate screen time to lower personal well-being and depressive [30] and anxious symptomatology [31].

On the contrary, people who reported an increase in the amount of time spent on hobbies presented higher self-esteem and better levels of flourishing. These results are consistent with previous studies that relate leisure activities with better mental health [32] and lower symptoms of depression [33] and anxiety [34] in the adult population. They are also related to recommendations made at the beginning of the pandemic to mitigate the possible negative effects of social distancing and quarantine [35].

In addition to the confinement and other restriction measures, many people have directly experienced the effects of COVID-19. Despite knowing someone with COVID-19 symptoms was a predictor of lower flourishing, those who indicated that they did not know anyone with symptoms or with a confirmed diagnosis had lower levels of flourishing than those who had a family member with symptoms. Also, knowing someone who had been hospitalized or someone who had died from COVID-19 were not related to flourishing levels. People who have not experienced effects on physical health firsthand, or through people close to them, might be more uncertain about the COVID-19 situation and might perceive the measures implemented as more restrictive and meaningless. Likewise, those who know people close to them with symptoms and confirmed diagnosis may feel optimistic about their improvement. A possible explanation for these results derives from the Terror Management Theory [36] which explains the conscious (proximal defenses) and unconscious (distal defenses) mechanisms that people use to protect themselves from death anxiety. Following this theory [8, 37], the higher levels of thriving of people who have family members with COVID-19 symptomatology could be distal defenses that decrease unconscious worry about their mortality through life purpose, better relationships, optimism, and improved self-esteem.

This study also provides predictive models for self-esteem, happiness, and flourishing. It was found that age, education, employment status, socioeconomic level, and time spent on hobbies predicted self-esteem. In the case of global happiness, only age, socioeconomic level and time spent on hobbies were significant predictors. Additionally, regarding flourishing, the significant variables were employment status, socioeconomic level, time dedicated to hobbies, and knowing a person diagnosed with COVID-19. The development of interventions aimed at maintaining and increasing the well-being of the population should consider these characteristics during their design and implementation.

### Conclusion and Limitations

Mental health is more than the absence of disease and the positive aspects of life might be more important than the absence of negative aspects [12]. Thus, this study focused on the positive aspects of mental health during the COVID-19 pandemic. Results showed that, in general, participants had high levels of self-esteem, considered themselves to be fairly or very happy individuals, and had an average level of flourishing. Age, education, socioeconomic status, time spent on mobile phones, and hobbies were found to be relevant to happiness, flourishing, and self-esteem.

Despite the relevance of this study, it is important to recognize its limitations. Although participation was high, having collected data through electronic means may limit the generalizability of the results. Future studies can expand the sample to obtain greater representativeness and include different methods of data collection. Likewise, caution should be exercised when interpreting the data due to their self-report nature. In addition, due to the cross-sectional nature of the study, we cannot establish causal relationships or know the possible longitudinal changes that may have occurred among participants. However, despite its limitations, we are confident that this study contributes to the understanding of aspects of mental health and well-being of the population during an unprecedented global emergency.

Future studies should take these results into account to expand these findings and propose prevention and intervention measures that contribute to promoting mental health by increasing self-esteem, happiness, and flourishing. Particular attention should be paid to the young population with lower levels of well-being. In addition, more should be known about leisure time and dedication to hobbies, which was the only variable that predicted the three constructs of interest. States, governments, and companies could benefit from our findings when analyzing and determining measures for social and economic reactivation.
